# Classification of Forefoot Plantar Pressure Distribution in Persons with Diabetes: A Novel Perspective for the Mechanical Management of Diabetic Foot?

**DOI:** 10.1371/journal.pone.0079924

**Published:** 2013-11-22

**Authors:** Kevin Deschamps, Giovanni Arnoldo Matricali, Philip Roosen, Kaat Desloovere, Herman Bruyninckx, Pieter Spaepen, Frank Nobels, Jos Tits, Mieke Flour, Filip Staes

**Affiliations:** 1 Department of Rehabilitation Sciences, Katholieke Universiteit Leuven, Leuven, Belgium; 2 Laboratory for Clinical Motion Analysis, University Hospitals Leuven, Pellenberg, Belgium; 3 Multidisciplinary Diabetic Foot Clinic, University Hospitals Leuven, Leuven, Belgium; 4 Department of Development & Regeneration, Katholieke Universiteit Leuven, Leuven, Belgium; 5 Department of Mechanical Engineering, Katholieke Universiteit Leuven, Leuven, Belgium; 6 Department of Internal Medicine- Endocrinology, Onze-Lieve-Vrouw Ziekenhuis Aalst, Aalst, Belgium; 7 Department of Dermatology, University Hospitals Leuven, Leuven, Belgium; Universidad Europea de Madrid, Spain

## Abstract

**Background:**

The aim of this study was to identify groups of subjects with similar patterns of forefoot loading and verify if specific groups of patients with diabetes could be isolated from non-diabetics.

**Methodology/Principal Findings:**

Ninety-seven patients with diabetes and 33 control participants between 45 and 70 years were prospectively recruited in two Belgian Diabetic Foot Clinics. Barefoot plantar pressure measurements were recorded and subsequently analysed using a semi-automatic total mapping technique. Kmeans cluster analysis was applied on relative regional impulses of six forefoot segments in order to pursue a classification for the control group separately, the diabetic group separately and both groups together. Cluster analysis led to identification of three distinct groups when considering only the control group. For the diabetic group, and the computation considering both groups together, four distinct groups were isolated. Compared to the cluster analysis of the control group an additional forefoot loading pattern was identified. This group comprised diabetic feet only. The relevance of the reported clusters was supported by ANOVA statistics indicating significant differences between different regions of interest and different clusters.

**Conclusion/s Significance:**

There seems to emerge a new era in diabetic foot medicine which embraces the classification of diabetic patients according to their biomechanical profile. Classification of the plantar pressure distribution has the potential to provide a means to determine mechanical interventions for the prevention and/or treatment of the diabetic foot.

## Introduction

The diabetic foot remains one of the most serious complications of diabetes mellitus [1]. Key pathophysiological factors are peripheral neuropathy, vasculopathy, non-enzymatic glycosylation of soft tissues and foot deformities [2]. The risk of foot ulceration can be increased because of alterations in the gait of persons with diabetes in combination with biomechanical changes of soft tissues [3]. Ulcerations are difficult to heal and often precede infection and lower extremity amputation [4]. Objective evaluation of gait alterations is therefore crucial as it can serve as a starting point for the development of treatment algorithms, preventive strategies and early detection [5]–[7].

Gait conditions associated to diabetes are most frequently assessed with plantar pressure measurement equipment because elevated pressures are considered as a major risk factor of ulceration in diabetic neuropathic feet with deformities [6], [8], [9]. Cross-sectional, comparative study designs are most commonly used and define populations on the basis of the presence or absence of diabetes, neuropathy, vasculopathy and history of ulceration (pathophysiological approach) [10]. Having provided valuable information on the pathomechanics of the diabetic foot, one may question its appropriateness for determining optimal redistribution/offloading strategies. An interesting alternative could be stratification of patients based on their plantar pressure pattern homogeneity (biomechanical approach). Such an approach may avoid the potential ‘smoothing’ of relevant pressure patterns inherently associated to the averaging methods adopted in pathophysiological studies [10]. Significant variations within a pathophysiological group are often reduced (smoothed), resulting in an inaccurate representation of pressure patterns seen across individuals. A biomechanical approach does not depart from the assumption of a linear relationship between a specific pathophysiological complication and pressure distribution patterns. Therefore, a biomechanical approach may embrace to a higher extend the general concept of plantar pressure distribution variability present in the so-called normal population [11]. Finally, reducing the four dimensional pedobarographic information of a clinical population to categorical data (e.g. clusters) may be attractive as it can serve as input for analytic and experimental studies using multivariable modelling techniques with a defined foot ulcer outcome [12]–[14].

The analysis of foot loading homogeneity introduces a new framework in diabetic foot medicine. Giacomozzi and Martelli [15] described a ‘functional classification’ and a ‘shape-based classification’ by considering barefoot plantar pressure measurements from diabetic cohorts and a control group. For the functional classification, the authors differentiated peak pressure curves based on the single or simultaneous occurrence of limited joint mobility, muscular weakness and increased peak pressure. Kmeans clustering algorithms applied on the magnitude and shape of the peak pressure curves were used to obtain a ‘shape-based classification’. Bennets et al [16] explored differentiation of regional peak plantar pressure in patients with diabetes using Kmeans clustering following total mapping of barefoot plantar pressure measurements into seven regions of interest. The authors obtained 2 to 10 clusters that were related to shoe wear design. Both previous mentioned research groups used Kmeans clustering algorithms for classification construction, but some limitations can be formulated. The ‘shape-based classification’ of Giacomozzi and Martelli [15] lacks discriminative value for diabetic foot ulceration diagnosis. Bennets et al [16] did not provide an in depth description of their study population (e.g. no in- and exclusion criteria reported), nor included a non-diabetic group. Inclusion of non-diabetic persons is to our opinion essential in the early stage of biomechanically oriented classification. This is considered important as, up-to-now, information is lacking to what extend patients with diabetes, without neuropathy or foot deformities, really differ from non-diabetic, age-related, persons. Knowledge of this can, in the future, serve as basis for research into prediction of for example foot ulcer development, further understanding of biomechanical factors related to the aetiology or design optimal intervention strategies. The present study originated from the premises of the potential benefits that plantar pressure pattern based classifications may yield with respect to: 1) the decision making process, 2) the communication between care takers involved in the decision making process or treatment, 3) treatment of diabetic patients.

The goal of the present study was therefore to study the classification of forefoot plantar pressure patterns among non-diabetic persons and diabetic patients through a non-hierarchical clustering technique. The rationale for specifically focussing on the forefoot relates to the development of plantar foot ulcers, of which approximately half develop under the metatarsal heads and hallux. Since gait classification is, in a primary stage, predominantly descriptive in nature, two specific aims were considered: i) exploring forefoot plantar pressure patterns of non-diabetic persons, diabetic patients and both groups together, ii) providing quantitative feedback with respect to the pattern construction. Next to these aims, two hypotheses were tested: i) patients with diabetes cannot be distinguished from non-diabetic peers based on their forefoot plantar pressure pattern, ii) forefoot plantar pressure based classification does not discriminate for other parameters not included for clustering selection.

## Research Design and Methods

### Subjects

Medical and clinical data collection as well as the analysis protocols was approved by the UZLeuven Medical Ethics Committee and written informed consent was obtained from each participant. Adults diagnosed with Type 1 and Type 2 diabetes, according to WHO criteria, were targeted in two Belgian Diabetic Foot Clinics (Flanders) (Univeristy Hospitals Leuven and Onze-Lieve-Vrouw Ziekenhuis Aalst). The Diabetic Foot Centres in the current study are both involved in the ‘Initiative for Quality Promotion and Epidemiology at Multidisciplinary Diabetic Foot Clinics’ organised by the The Scientific Institute for Public Health (Belgium). A specific registry has been created since four years, which aims at systematically reporting medical information with respect to diabetic foot ulcers. On this basis, the diabetic foot centres are archiving the medical history and ‘diabetic foot history’ in a concise and similar way. Before the recruitment of the diabetic patients began, different members from the hospital based diabetic foot teams participated actively in the design of the study. A consensus meeting was organised in order to agree on a study-specific medical record and standard physical screening.

The total diabetic population recruited in the two diabetic foot clinics consisted of 97 adults. Recruitment started in both clinics in 2010 and lasted for one complete year. Inclusion criteria for the subjects with diabetes were: age between 45–70 years, walking without walking aids, BMI between 20 kg/m^2^ and 40 kg/m^2^, oedema score<2 [17], no active foot ulcer or amputation, no history of orthopaedic lower limb surgery and no Charcot neuroarthropathy. Following recruitment in each clinic, a study-specific medical record and standard physical screening were completed.

Collected information from the medical record was related to 1) diabetes (e.g. duration, treatment), 2) laboratory assessment of blood samples (e.g. metabolic control past 6 months (HbA1c), creatinin level), 3) complications associated to diabetes (e.g. cardio-vascular status, visual impairment, history of ulceration). The physical examination included: 1) assessment of vibration sensation with 128-Hz tuning fork, 2) assessment of cutaneous pressure perception (10 g Semmes Weinstein monofilament, six sites), 3) palpation of dorsalis pedis and posterior tibial pulses of both feet, 4) determination of the foot deformity score for both feet [18] (prayer sign excluded), 5) passive range of motion measurements (e.g. hallux, ankle).

In addition to the diabetic group, thirty-three non-diabetic persons were recruited through advertisement at the Univeristy Hospitals of Leuven. Inclusion criteria for this group were: age between 45–70 years, BMI between 20 kg/m^2^ and 40 kg/m^2^, no history of orthopaedic lower limb surgery or injury, absence of any known neurological or systemic disease.

### Instrumentation and Gait Analysis Protocol

Gait analysis of all recruited individuals was performed in the Laboratory for Clinical Movement Analysis of the Univeristy hospital Leuven using the following measurement devices: a 3D motion analysis system, a plantar pressure platform and a force platform. The passive motion analysis system (Vicon Motion System Ltd, Oxford Metrics, UK) consisted of 10 T-10 cameras surrounding a 10 m walkway in order to track kinematic data (100 Hz) of all participants. In the aforementioned walkway, a custom made force plate was placed in the middle (Advanced Mechanical Technology, Newton, MA,US) covered with a pressure plate (dimensions 0.5 m×0.4 m, 4096 resistive sensors, spatial resolution 2.8 sensors per cm^2^, RSscan International, Olen, Belgium). A second force plate was also embedded in the walkway, aligned with the custom made force plate. This set-up allowed the detection of specific gait events as well as a continuous calibration of the pressure plate with the AMTI force plate using the so-called 3-D box calibration interface (RSscan International, Olen, Belgium). Time synchronization between the pressure plate and motion analysis system was achieved by measuring the optimal signal correlation between the force signals of both pressure and force plate [19]. Data from the force plate and pressure plate were sampled at 200 Hz.

Dynamic barefoot plantar pressures were measured with individuals walking at a self-selected speed until five ‘representative’ walking trials were recorded. A trial was considered representative if the participants made clear pedobarograph contact with good inter-trial consistency, judged by visual inspection of an experienced researcher. The current set-up allowed using the midgait protocol for all individuals [20]. All gait analyses were performed by one experienced clinician. Temporal-spatial parameters of all gait cycles were determined based on input of force plate data (AMTI) together with the identification of gait events within 3D motion analysis software.

### Data Analysis

**Figure 1:**
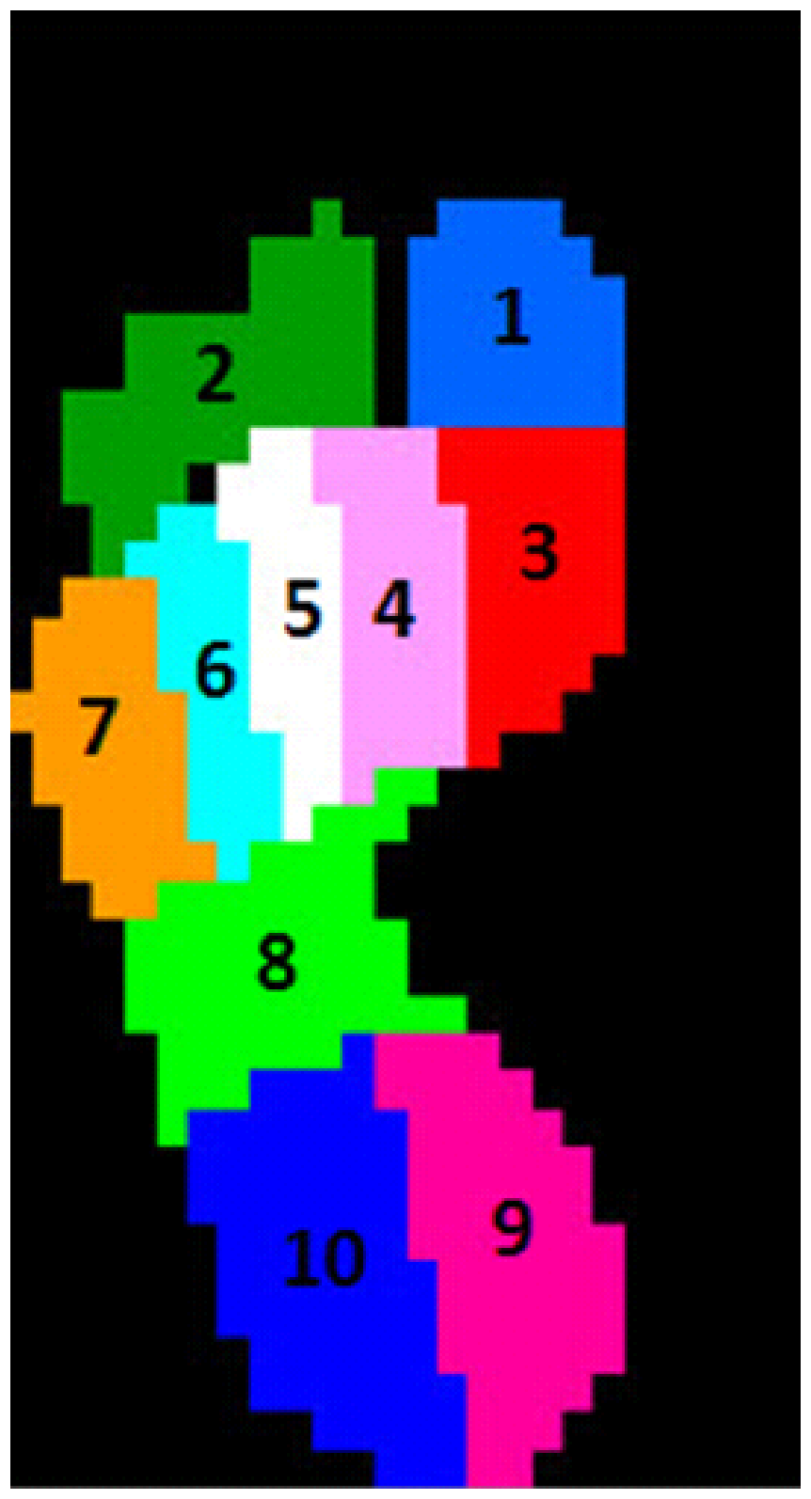
Total mapping technique applied in the current study. Illustration of the ten regions of interest where semi-automatically defined on the peak pressure footprint. The regions of interest were 1) hallux, 2) toes 2–5, 3) first metatarsal, 4) second metatarsal, 5) third metatarsal, 6) fourth metatarsal, 7) fifth metatarsal, 8) midfoot, 9) medial heel, 10) lateral heel.

Footscan 7.97 gait 2^nd^ generation (RSscan International, Olen, Belgium) was used to analyse the pressure data. A semi-automatic total mapping method was applied to identify ten regions of interest on the peak pressure footprint of each trial. The regions of interest were: hallux (T1), toes two to five (T2–5) considered as one region, the individual metatarsal heads (MTH) one to five (MTH1-5), midfoot (MF), medial heel (HM) and lateral heel (HL) (figure 1). The reliability of the aforementioned mapping method has recently been evaluated [21]. Through a repeated measures design, this method was found to have negligible inter-therapist variability.

Following semi-automatic total mapping, force-time integral and maximum of the peak force sensor was extracted for all regions of interest, except for the midfoot and toes two to five. Subsequently, relative regional impulses (RrI, as % of summed impulses) were calculated considering the remaining eight regions of interest. Average RrI were calculated based on all trials of each individual in order to obtain one profile for each person and each foot (left and right foot were kept separated). The above mentioned approach is similar to that of De Cock et al [22].

### Statistical Analysis

Kmeans clustering was used to classify the RrI of the forefoot (the five metatarsals and the hallux). The approach described by Sawacha et al [10] was adopted in the current study. In a first phase, the RrI of the forefoot were converted into z-scores. Subsequently, a Kmeans function (Matlab 2012a; The Mathworks, Natick, US) was used and a standard Euclidean distance was selected for the partitioning into clusters. Since the iterative Kmeans algorithm uses randomly generated starting points in an optimization scheme, all Kmeans calculations were repeated 10 times, and the best outcome, also called ‘criterion of best’, was taken as the position of the cluster centres. Ten repetitions ensured repeatable results (identical cluster centres for multiple runs of 10) as already mentioned by other authors [22], [24]. The decision making process for the optimal number of clusters (classification construction), was supported by calculating the average silhouette coefficient (SC) for each chosen number of clusters. The following formula was used for determining the SC:
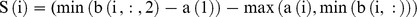



With *a(i)* being the average distance from the ith point to the other points in its cluster, and *b (I,k)* being the average distance from the ith point to points in another cluster k. The value of S(i) ranges from −1 to  = 1. A value close to +1 indicates a good clustering, a value close to −1 indicates that the assignment is probably to the wrong cluster. Finally, the SC was calculated by considering the average of all *S (i)* for a given k clustering. The aforementioned calculation was repeated 10 times for each k clustering, and the highest SC was considered as the most representative classification. We adopted the minimum benchmark of 0.25 for adopting a classification system [23].

The clustering process, including the determination of the optimal number of clusters, was consecutively performed for the control group (CtrlOnly, number of feet = 66), the diabetic group (DbtOnly, number of feet = 194) and finally for both groups together (BothGr, number of feet = 260). Results of these three explorations were subsequently evaluated in a qualitative way.

Inferential statistical analyses to reject/accept the hypotheses were conducted on the outcome measurements from the optimal clustering performed on both groups together (BothGr). First normality of the data was evaluated by plotting normal probability plots as well as by performing Kolmogorov-Smirnov tests. One-way ANOVA was used to determine statistical differences between different clusters when dealing with interval data. Fisher-Freeman-Halton test was performed to detect significant differences between nominal data of each cluster. Finally, a Kruskal-Wallis test was applied to the ordinal data of the same groups. Both non-parametric tests were used as data proved to be non-normally distributed. When appropriate, Tukey-Kramer or Fischer’s exact test was performed to complete the multiple comparison procedure. Bonferroni procedures were used for all post-hoc analyses. All statistical calculations were performed within Matlab 2012a (The MathWorks, Inc., Natick, US).

## Results

**Figure 2:**
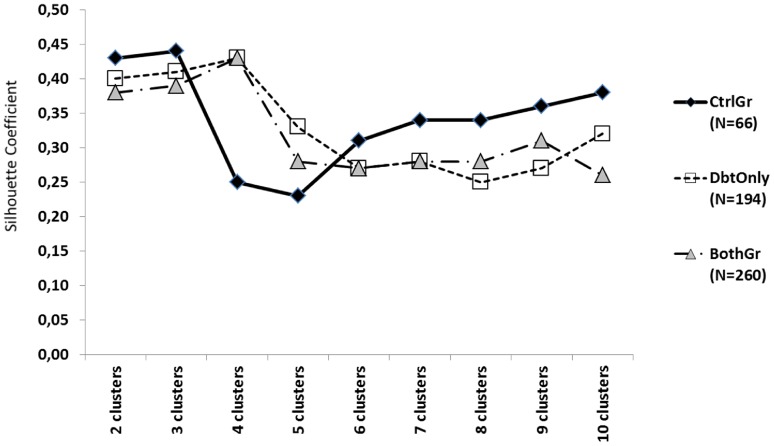
Summary of silhouette coefficient (SC) calculations considering k values between 2 and 10. Decision making process regarding ‘optimal’ number of clusters was performed on the basis of this graph.

**Figure 3:**
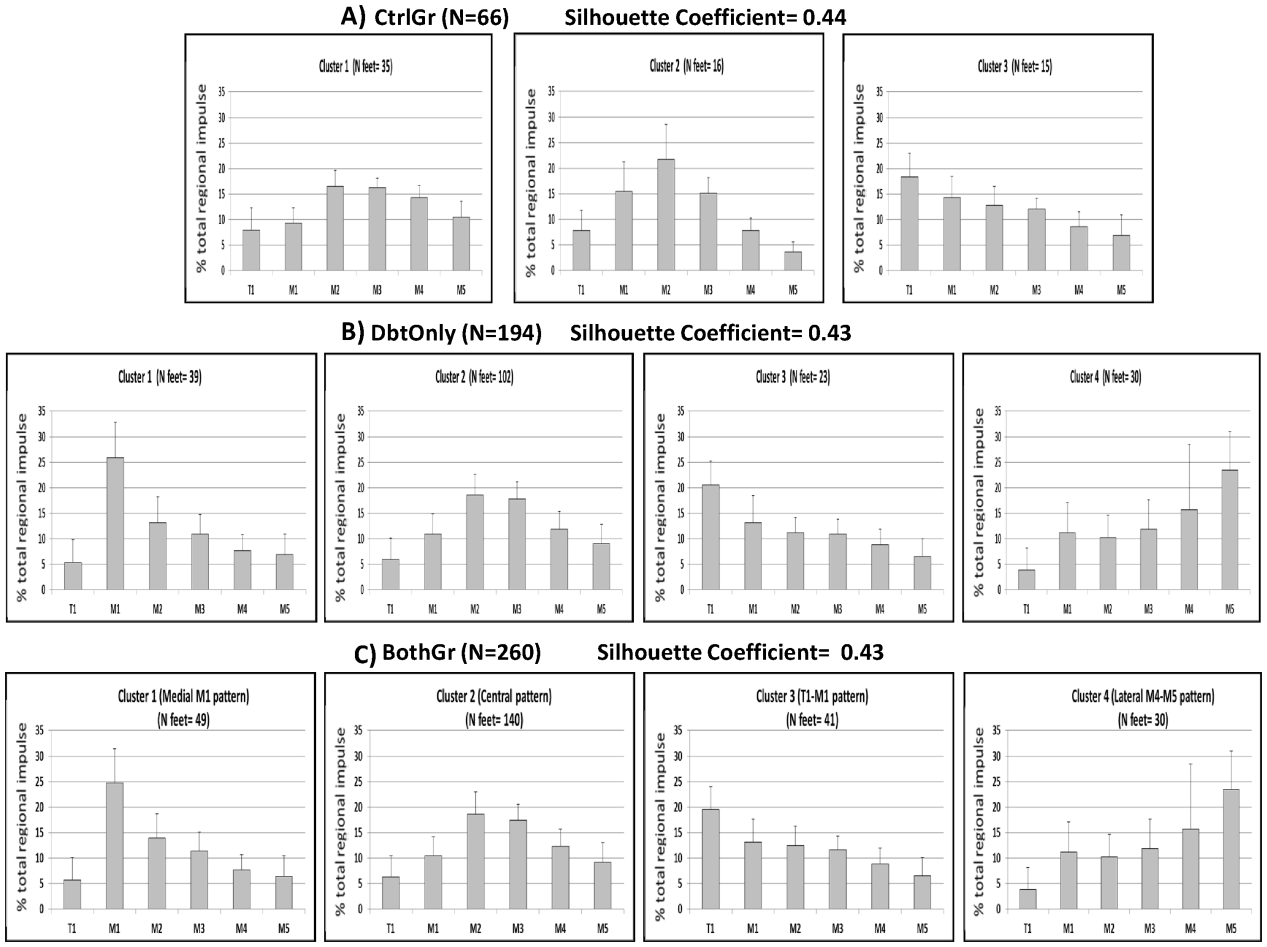
Summary of classification construction. A) RrI for the forefoot segments of the three loading patterns considering only data of control group, B) RrI for the forefoot segments of the four loading patterns considering only data of diabetic group, C) RrI for the forefoot segments of the four loading patterns considering data from both groups.

Results for determining the adequate number of clusters are shown in figure 2. Three clusters were most suitable when considering only RrI of CtrlOnly (SC = 0.44). The preferred number of clusters was four (SC = 0.43) for DbtOnly, which was also the case for BothGr (SC = 0.43). Figure 3 (a,b,c) provides a summary of the plantar pressure loading patterns for each cluster.

The first cluster of CtrlOnly showed a scattered forefoot loading (figure 3a), the second cluster was characterized by a major loading at the central MTH whereas the third cluster suggested a progressive higher loading from the lateral to the medial segment of the forefoot.

The clusters for the DbtOnly had four distinct patterns (figure 3b). The first cluster was characterized by a distinct loading of the first metatarsal head, whereas the second and third cluster showed good resemblance to clusters 1 and 3 of the CtrlOnly clustering. Cluster four was characterized with a much more lateral oriented forefoot loading.

**Figure 4:**
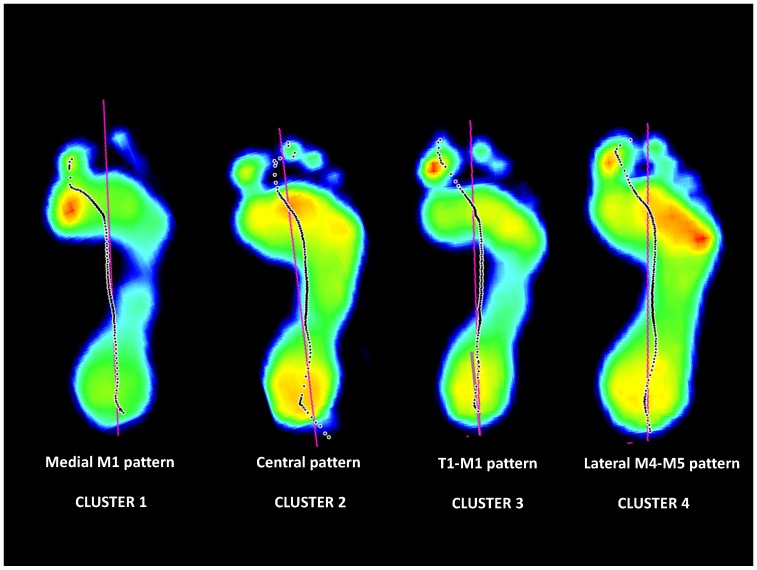
Example of a peak pressure footprint for each cluster. Selected footprints are coming from the diabetic group.

Finally, forefoot loading patterns clustering based on data of BothGr revealed two remarkable observations (figure 3c). First, good resemblance was observed between the optimal clustering for BothGr and the DbtOnly clustering. Second, 100% of the feet in cluster four of BothGr were from persons with diabetes (figure 3c, Table 1). Based on the results, it was decided to use the optimal clustering based on the BothGr data. Following cluster names were introduced: cluster 1 = Medial M1 pattern, cluster 2 = Central pattern, cluster 3 = T1-M1 pattern, cluster 4 = Lateral M4–M5 pattern. An example of a peak pressure footprint for each cluster is provided in figure 4.

**Table 1:** Summary of descriptive characteristics for classification system based on data of both groups. Data have been separated for controls and diabetics.

		Medial M1 pattern Cluster 1		Central Pattern Cluster 2		T1-M1 pattern Cluster 3		Lateral M4–M5 pattern Cluster 4		p value	
		Diabetics	Controls	Diabetics	Controls	Diabetics	Controls	Diabetics	Controls		
	Age (years)	63.6 (7.9)	54.6 (4.8)	59.3 (9.4)	55.6 (6.7)	63.1 (7.9)	51.7 (6.3)	65.4 (7.9)	/	<0.0001	
	Number of feet	41	8	99	41	24	17	30	/		
	% Bilateral	19.5	50	68.5	83	25	70	33.5	/		
	% Unilateral	80.5	50	31.5	17	75	30	66.5	/		
	Height (cm)	171.6 (8.1)	170.1 (9.9)	169.7 (10.1)	173.1 (8.4)	174.4 (8.6)	169.4 (8.9)	174.4 (9.3)	/	0.1414	
	Weight (kg)	86.2 (15.3)	74.1 (17.4)	82.6 (16.3)	76.2 (16.1)	88.9 (17.1)	66.1 (13.1)	88.9 (15.8)	/	0.0385	
	Body Mass Index	29.2 (4.7)	25.3 (3.4)	28.5 (4.5)	25.2 (3.7)	29.2 (4.2)	22.8 (2.6)	29.3 (5.4)	/	0.0542	
History of foot ulcer	History of foot ulcer(N = Feet)	8	/	12	/	5	/	6	/		
	Toe ulcers (includeshallux) (dorsal)	4	/	4	/	1	/	1	/		
	Lesser toe ulcers(plantar)	2	/	1	/	–	/	–	/		
	Hallux Ulcer(plantar)	2	/	2	/	3	/	1	/		
	First metatarsalhead(plantar)	1	/	2	/	–	/	1	/		
	Third metatarsalhead(plantar)	–	/	1	/	–	/	–	/		
	Fifth metatarsalhead(plantar)	–	/	–	/	–	/	3	/		
	Heel ulcer (plantar)	2	/	1	/	–	/	–	/		
	Heel ulcer (posterior)	–	/	–	/	–	/	1	/		
	Midfoot (dorsal)	–	/	1	/	1	/	–	/		
	Midfoot (plantar)	–	/	1	/	–	/	–	/		

Where appropriate, mean and standard deviation is provided. Inferential statistics were performed on cluster level. Significant changes with respect to age observed between cluster 1 and 2, 2 and 4 as well as 3 and 4.

The total number of diabetic feet clustered in the lateral M4–M5 pattern was thirty (Table 1). Only five persons with diabetes were stratified in this pattern with both feet, whereas the other 20 feet where unilateral patterns. This trend towards a unilateral forefoot loading pattern was observed in all clusters, and was much more prominent in persons with diabetes (Table 1). The medical information showed that all patients with a history of a plantar foot ulcer at the fifth MTH (N = 3) were stratified in the lateral M4–M5 pattern. Similarly, all plantar ulcers (N = 3) observed in the T1-M1 pattern were located under the hallux (Table 1).

The number of feet was, for women, significantly different in cluster 3 when compared to cluster 2 (Table 2) (p = 0.000912). Significant differences within the number of diabetic feet were observed between clusters 2 and 3 compared to cluster 4 (p = 0.00004) (Table 2). No other significant differences could be observed within the medical information of the plantar pressure based clustering. Approximately 75% of the diabetics in clusters 1 and 4 had a risk category higher then 1, whereas in clusters 2 and 3 this was about 50%. A slower walking speed was observed in cluster 4 (p<0.01), whereas the other three clusters where characterized by a quite similar speed (Table 3). One-way ANOVA analyses showed significant differences between the four clusters for RrI and Peak Force. These significant differences were present in a distinct majority of the regions of interest (Table 3). The loading under the fourth and fifth MTH was significantly higher in the lateral M4–M5 pattern compared to the other three groups. The loading under the first MTH was the highest in the Medial M1 pattern, whereas the Central pattern cluster was characterized by significantly higher loading under the third and fourth MTH. Finally, the T1-M1 pattern distinguished significantly with respect to the other groups at the level of the loading of the hallux.

**Table 2:** Overview of clinical parameters associated to the diabetic patients of each cluster (based on kmeans clustering considering data of both groups).

	Medial M1 pattern Cluster 1	Central Pattern Cluster 2	T1-M1 pattern Cluster 3	Lateral M4–M5 pattern Cluster 4	p value
Feet of Men	29	55	22	25	0.000912*
Feet of Women	12	44	2	5	
Number of diabetic feet	41	99	24	30	0.00004**
Number of non-diabetic feet	8	41	17	0	
Diabetes Type 1	13	35	2	5	0.02475
Diabetes Type 2	28	64	22	25	
Risk category 0	10	49	12	8	0.6005
Risk category 1	2	2	1	1	
Risk category 2a–b	4	9	5	6	
Risk category 3	25	39	6	15	
Diabetes duration (years) (average/stdev)	20.4 (13.3)	16.1 (11.5)	16.0 (10.6)	21.1 (10.5)	0.0592
History of foot ulcers	8	12	5	6	0.587062
No history of foot ulcers	33	87	19	24	
Sens Monofil 10 g (0/6)	7	11	8	6	0.1801
Sens Monofil 10 g (1/6)	3	5	2	5	
Sens Monofil 10 g (2/6)	5	2	2	3	
Sens Monofil 10 g (3/6)	3	3	0	2	
Sens Monofil 10 g (4/6)	2	4	0	1	
Sens Monofil 10 g (5/6)	6	15	2	2	
Sens Monofil 10 g (6/6)	15	59	10	11	
Pedal Pulses (0/2)	8	9	0	5	0.2322
Pedal Pulses (1/2)	10	23	3	6	
Pedal Pulses (2/2)	23	67	21	19	
FDS 0/4	10	50	10	7	0.0909
FDS 1/4	15	19	7	10	
FDS 2/4	9	14	2	4	
FDS 3/4	5	12	3	3	
FDS 4/4	2	4	2	6	

p value following Bonferroni correction = 0.05/26 = 0.002, *Pairwise Fisher exact test for number of diabetic feet: cluster 2 and 3 significantly different, **Pairwise Fisher exact test for feet men/women: cluster 2 significant different from 3 and 4, FDS: Foot Deformity Score: 6 point scale (1 point for each characteristic: small muscle wasting, bony prominence, prominent metatarsal heads, hammer/claw toes, limited joint mobility, charcot foot deformity). (Charcot foot was an exclusion criteria for this study, limited joint mobility not considered here (prayer sign)). Pedal pulses: palpation of the dorsalis pedis and tibial pulses. Sens Monofil 10 g: sensation of the 10 g monofilament (6 point scale per foot). Risk classification based on Belgian guidelines: risk category 3 = Diabetic patient with at least one of the following complications: history of ulceration/peripheral arterial disease, risk category 2b: = diabetic patient with neuropathy and pronounced rigid foot deformities, risk category 2a = diabetic patient with neuropathy and mild/flexible foot deformities, risk category 1 = diabetic patient with neuropathy, risk 0 = diabetic patient without complications as mentioned in other risk categories.

**Table 3:** Summary of temporal, spatial and pressure related data of each cluster based on kmeans clustering considering data of both groups.

		Medial M1 pattern Cluster 1	Central Pattern Cluster 2	T1-M1 pattern Cluster 3	Lateral M4–M5 pattern Cluster 4	p value
Temporal-spatialparametersof gait	Cadence (steps/min)	105.2 (13.4)	108.6 (12.4)*^4^	108.2 (10.4)	99.8 (13.4)*^2^	<0.01
	Stance_Time (% gait cycle)	61.6 (3.2)	60.6 (2.5)*^4^	61.1 (3.2)	62.3 (4.6)*^2^	<0.01
	Swing_Time (% gait cycle)	38.4 (3.2)	39.4 (2.5)*^4^	38.9 (3.2)	37.6 (4.6)*^2^	<0.01
	Walking_Speed (m/s-1)	1.0 (0.3)	1.1 (0.2)*^4^	1.1 (0.1)*^4^	0.9 (0.2)*^2,3^	<0.001
% totalregionalimpulse	HL	14.3 (4.1)*^2,4^	11.8 (3.7)*^1^	13.2 (3.0)	11.5 (4.3)*^1^	<0.001
	HM	15.9 (5.0)*^2,4^	13.8 (3.5)*^1^	14.8 (3.1)*^4^	12.3 (4.5)*^1,3^	<0.001
	T1	5.7 (4.3)*^3^	6.3 (4.2)*^3,4^	19.6 (4.5)*^1,2,4^	3.8 (4.4)*^2,3^	<0.001
	M1	24.4 (6.6)*^2,3,4^	10.4 (3.7)*^1,3^	12.9 (4.4)*^1,2^	11.1 (5.9)*^1^	<0.001
	M2	13.9 (4.9)*^2,4^	18.6 (4.5)*^1,3,4^	12.5 (3.8)*^2^	10.2 (4.5)*^1,2^	<0.001
	M3	11.5 (3.7)*^2^	17.5 (3.0)*^1,3,4^	11.8 (2.5)*^2^	11.9 (5.8)*^2^	<0.001
	M4	7.8 (2.9)*^2,4^	12.3 (3.4)*^1,3,4^	8.9 (3.1)*^2,4^	15.7 (12.8)*^1,2,3^	<0.001
	M5	6.5 (4.1)*^2,4^	9.2 (3.7)*^1,3,4^	6.4 (3.5)*^2,4^	23.5 (7.6)*^1,2,3^	<0.001
Peak Force(Newton)	HL	17.1 (4.5)	16.5 (3.0)	16.8 (4.1)	15.8 (2.9)	n.s.
	HM	19.2 (5.4)*^4^	17.8 (3.6)	18.5 (5.1)	16.0 (2.6)*^1^	0.02
	T1	19.4 (12.0)*^3^	19.0 (8.4)*^3^	32.2 (12.8)*^1,2,4^	18.4 (12.2)*^3^	<0.001
	M1	29.7 (10.4)*^2,3,4^	13.2 (5.7)*^1^	16.8 (8.9)*^1^	17.7 (10.0)*^1^	<0.001
	M2	17.7 (7.7)*^2^	22.1 (6.9)*^1,3,4^	14.5 (5.8)*^2^	16.0 (5.9)*^2^	<0.001
	M3	14.1 (5.0)*^2^	19.6 (5.8)*^1,3,4^	12.8 (4.1)*^2^	16.0 (5.9 )*^2^	<0.001
	M4	9.3 (3.3)*^2,4^	13.0 (3.5)*^1,3,4^	9.3 (3.2)*^2,4^	15.9 (5.1)*^1,2,3^	<0.001
	M5	8.7 (7.0)*^4^	10.7 (5.5)*^4^	7.1 (3.5)*^4^	28.8 (12.2)*^1,2,3^	<0.001

Provided data represent averages together with standard deviation. n.s. = not significant.

## Discussion

We explored the classification of forefoot plantar pressure distribution in diabetics using an unsupervised learning technique. The step-by-step approach used in this study, suggests that a biomechanical classification of diabetic foot may provide a basis for further research in aetiology, prediction and treatment of foot related problems in patients with diabetes. Most clusters contained persons from both the control group as well as the diabetic group. Only the lateral M4–M5 pattern cluster consisted of persons with diabetes only. These findings suggest the need for other approaches to investigate the biomechanical profile rather than a purely pathophysiological-based approach (e.g. neuropathy).

The results for CtrlGr in the current study show good face validity with published data from De Cock et al [22]. Cluster one from the current study is similar with their ‘Central pattern’, whereas the properties of clusters 2 and 3 are similar to respectively their ‘M2 pattern’ and ‘Medial M1 pattern’ [22]. De Cock et al [22] described one additional cluster. One possible explanation for this difference may be our quantitative method for optimal clustering, whereas De Cock et al [22] based their gait classification on research by other authors. Other reasons may be the considerable difference in age and the fact that participants in the study of De Cock et al [22] were running.

The stratified clusters following the Kmeans clustering for BothGr show agreement with established clinical concepts. The T1-M1 pattern for example, characterized by a high RrI at the hallux, has typically been associated to sagittal plane dysfunction of the hallux [25]. Limitation of dorsiflexion motion at the hallux during terminal stance, either with a structural or functional aetiology, impedes the adequate transfer of loading between the first metatarsal and hallux. This (patho)mechanical manifestation is not only related to diabetes induced limited joint mobility, it has also been reported in non-diabetics. A similar profile has been visualized by Bennets et al [16]. These authors described this pattern as a pattern containing a group of persons with high hallux pressures. Our Medial M1 pattern, characterized by a high RrI at the first metatarsal head, can be compared with one of the groups reported by Bennets et al [16]. In our study, only eight feet from control subjects were stratified into this cluster making it a diabetic population dominant cluster (Table 1). Plantar flexed position of the first metatarsal, fat pad atrophy, forefoot valgus and turf toe are some of the factors that clinically can be related to such an important temporal loading of the first metatarsal. In persons with diabetes, this may originate from motor neuropathy (e.g. tibialis anterior weakness, intrinsic muscle denervation) as well as from chronic trauma of the insensate foot. The Central pattern of BothGr can be compared with the third group of Bennets et al [16] following clustering with k set at 5 (found in 67 feet/819). From a mechanical viewpoint, this loading pattern has been linked to the important weight-bearing function of the second metatarsal and its restricted mobility at the Lisfranc joint [26]. Other potentially contributing factors to this loading pattern are first ray insufficiency, fat pad atrophy and clawing of toes [27].

The Medial M1 pattern, Central pattern and the T1-M1 pattern show good face validity with published data originating from so-called normal participants. The lateral M4–M5 pattern is a profile that, from a clinical viewpoint, cannot be considered as ‘typical’. It illustrates the poor contribution of the medial column of the forefoot to the overall weight bearing function of the forefoot. In an attempt to cross-validate this pattern, one might compare it with the pattern associated to group 5 with k = 7 from Bennets et al [16]. This group, composed of 65 feet (out of 819), was characterized by a higher peak pressure at the middle (metatarsal 2–4) and lateral (fifth metatarsal) segment of the forefoot whereas low pressures were described under the first metatarsal and hallux. The lateral M4–M5 pattern in the present study only consisted of diabetics. Sawacha et al [10] also isolated three ‘pure’ diabetics groups based on applying Kmeans clustering algorithms on 3D lower limb model kinematics and kinetics. Though, the present study did not consider kinematics.

The biomechanical approach of the present study for stratification of persons with diabetes, is totally different from the well-accepted approach to classify diabetics on the presence of neuropathy, BMI and age, prior to initiating biomechanical comparisons. The current study unravels an innovative perspective to the diabetic foot community, while, it has the potential to meet all the criteria put forward by the International Working Group on the Diabetic Foot regarding classification systems. This expert group postulated that a classification system for clinical practice should facilitate communication between clinicians, support the decision making process and provide information about the healing potential of an ulcer [28]. The obtained classification in the present study, may facilitate communication as it starts from the homogeneity of plantar pressure patterns and results suggest that there might be a relationship between pattern and (past of) presence of plantar foot ulcers. The new classification might potentially enhance the decision making process, more particular decisions needed to be taken with regard to the most optimal offloading or redistribution strategy for a specific biomechanical group or ‘cluster’. The authors do recognise that further, prospective, studies are necessary to further investigate the multidimensional aspect of developing an ulcers starting from the new classification and to investigate the effects of treatment on load patterns of the foot. They suggest to first stratifying the recruited population on the basis of plantar pressure parameters, before installing an interventional plan. Ultimately, this may result in the development of ‘cluster’-specific guidelines for CAD/CAM fabricated foot orthoses. Finally, prospective studies will have to be conducted to evaluate the healing potential and/or preventive value of newly developed guidelines and treatments.

Another dilemma which should be addressed in the future comprises aspects where foot ulcer location does not coincide with an ‘expected’ pressure pattern. For example, one could raise the question what to do with a patient who belongs to cluster four and has an ulcer at the plantar aspect of the hallux. At this stage, it is reasonable to assume that this is highly speculative, as it is unknown if the pressure pattern of the patient has changed over time or not. Currently, applying an off-loading technique which redistributes the pressure maximally over the total plantar aspect of the foot (thus also some reduction under the M4–M5 region) and which reduces the pressure under the hallux extensively (typically −90%) would be the intervention of first choice. Thus, correlating foot ulcer location with pressure pattern will be mandatory in the future, as this will help in determining whether ‘the biomechanical approach’ has superior features compared to the ‘pathophysiological approach’ when it comes to foot ulcer prognosis.

Dobson et al [29] instructed that gait classification studies should report their supposed strengths and limitations. The strengths of the current study are its cross-sectional character, the multi-centre recruitment, the transparent reporting of the decision making process with respect to classification construction, the inclusion of a control group and the inclusion of medical parameters. Relative regional force-impulses from specific forefoot regions were purposely chosen in the current study. The main reason for focussing on the forefoot is that half of the plantar foot ulcers are located under the metatarsal heads and hallux [30]–[33]. An increase of peak pressures in these areas is one of the first ‘clinical’ observations in the absence of any clinically detectable neuropathy [33]. A recent study has highlighted that pedobarographic data originating from the forefoot are most relevant in detecting high risk patients [34]. Closely related to the selection of a specific subsampling of the footprint, is the choice of specific force/pressure related quantities which can be force-, time- or surface-dependent. In the current study, RrI were considered as it allows comparison between foot parts and between individuals [35] and, as such, overcome the lack of absolute reference values that researchers and clinicians are typically facing when using plantar pressure quantities. In this perspective, it is worth considering the role of body weight and walking speed on the classification process. It has been assumed in literature that plantar pressures are affected by these two parameters. With respect to walking speed this correlation has been clearly demonstrated [36]–[38], however, with respect to body weight some debate still exists [39]. Looking closer to the data in table 1 and 3 highlights some differences between the four clusters for these two factors. In an additional analysis (results not shown) we compared the results from the ANOVA tests, with the result of an ANCOVA where the same differences were evaluated after correction for walking speed and body weight. The results clearly indicated that the differences in relative pressure between the clusters are not due to the differences in walking speed and body weight. Only for metatarsal head two and three (M2 and M3) walking speed turned out to be only significant, whereas body weight was not related at all with relative pressure in the ANCOVAs. More important, the magnitude of the differences between clusters remained comparable (and significant at p<0.0001) in the analyses with and without correction for both covariates.

A limitation of the current study may be the sample size of both study cohorts. However, review of the literature has illustrated that it is not common practice to perform a priori sample size estimation. One of the major reasons for this lack relates to the fact that gait classification is often explorative in nature. Furthermore, performing post-hoc power analyses is, despite being highly attractive, subject to considerable debate in the literature. Qualitative evaluation of the sample size used in a certain gait classification study is often indirectly done through (cross-) validation studies. The main objective of such studies is to provide an adequate picture of the clinical and research applicability of the proposed classification system. The underlying principles in such studies can be diverse: evaluating robustness, internal validity and reproducibility. Thus, additional recruitment of new participants (both diabetic persons and so-called healthy controls) and evaluate its effect on the described classification system should be one of the next steps. The potential benefits highlighted in our discussion are at this point hypothetical. For example, the potential benefit of the proposed classification to unloading therapies, can be cross-validated through comparisons with in-shoe measurements. This can be an interesting future research topic. Another limitation of the current study is the fact that the impact of sex and left/right foot asymmetry has not been considered in the current study. Both elements have been evaluated by De Cock et al [22] who found, especially for the heel region, a considerable asymmetry. Finally, it should be stressed that Kmeans clustering is not the only available method for classification construction. Dobson et al [29] distinguished qualitative and quantitative strategies for gait classification construction. Whereas in the past, typically qualitative pattern recognition techniques were used to classify gait, nowadays, quantitative methods are predominantly used. Qualitative construction methods involve decisions made by group members and has lost popularity due its high subjectivity. Contrarily, quantitative methods encompass well-known and generic methods to analyse gait parameters (e.g. principal component analysis, neural networks, wavelet transformation, self-organizing maps, Bayesian networks,…). For these quantitative methods, the literature provides some guidelines to aid selection, but as a rule of thumb, it is recommended using different methods to same data and chose those that give the most useful solutions.

## Conclusion

A new era seems to emerge in diabetic foot medicine which encompasses the classification of patients with diabetes according to their biomechanical profile. The adoption of this alternative model has the potential to provide better management of the diabetic foot. The dimensions related to this alternative approach are multiple and the scientific community is facing many challenges if clinical significant results are pursued. Defining the most optimal number of groups or ‘clusters’, on one hand, and leaving the concept of ‘normality’ [11] on the other hand, are two examples of such challenges.
